# Robot-Assisted PSMA-Radioguided Salvage Surgery for Oligorecurrent Prostate Cancer Using the Novel SENSEI^®^ Drop-in Gamma Probe: Correlation of Intraoperative Measurements to Preoperative Imaging and Final Histology

**DOI:** 10.3390/cancers17010093

**Published:** 2024-12-31

**Authors:** Giovanni Mazzucato, Fabian Falkenbach, Marie-Lena Schmalhofer, Farzad Shenas, Maria Angela Cerruto, Alessandro Antonelli, Pierre Tennstedt, Markus Graefen, Felix Preisser, Philipp Mandel, Sophie Knipper, Lars Budäus, Daniel Koehler, Tobias Maurer

**Affiliations:** 1 Martini-Klinik Prostate Cancer Center, University Hospital Hamburg-Eppendorf, 20246 Hamburg, Germany; dott.giovannimazzucato@gmail.com (G.M.); f.falkenbach@uke.de (F.F.); p.tennstedt@uke.de (P.T.); graefen@uke.de (M.G.); f.preisser@uke.de (F.P.); p.mandel@uke.de (P.M.); sophieknipper@hotmail.de (S.K.); budaeus@uke.de (L.B.); 2 Department of Urology, Azienda Ospedaliera Universitaria Integrata di Verona, University of Verona, 37126 Verona, Italy; mariaangela.cerruto@univr.it (M.A.C.); alessandro.antonelli@univr.it (A.A.); 3 Department of Diagnostic and Interventional Radiology and Nuclear Medicine, University Medical Center Hamburg-Eppendorf, 20246 Hamburg, Germany; m.schmalhofer@uke.de (M.-L.S.); f.shenas@uke.de (F.S.); d.koehler@uke.de (D.K.); 4 Department of Urology, Vivantes Klinikum am Urban, 10967 Berlin, Germany; 5 Department of Urology, University Hospital Hamburg-Eppendorf, 20246 Hamburg, Germany

**Keywords:** biochemical recurrence, metastasis-directed therapy, minimal invasive, detection rate, CPS count

## Abstract

Background: To examine the feasibility and safety of the SENSEI^®^ drop-in gamma probe for robot-assisted, prostate-specific membrane antigen (PSMA)-radioguided salvage surgery (RGS) in lymph node or local oligorecurrent prostate cancer (PCa), detected via PSMA positron emission tomography/computed tomography (PET/CT). Methods: The first thirteen patients with pelvic oligorecurrent PCa who underwent [^99m^Tc]Tc-PSMA-I&S RGS using the SENSEI^®^ drop-in gamma probe at the Martini-Klinik (February–June 2024) were retrospectively analyzed. Radioactivity measurements in counts per second (CPS) as absolute values or ratios (CPS of tumor specimens/mean CPS from the patients’ benign tissues) were correlated with preoperative imaging and pathological findings (benign/malignant, lesion size). Postoperative complete biochemical response (cBR) was defined as prostate-specific antigen (PSA) levels of <0.2 ng/mL. Results: Fifty-four specimens were removed from 13 patients, with nineteen (35%) containing PCa. All patients had one PSMA PET/CT-positive lesion, which were all detected intraoperatively. These lesions showed higher ex vivo CPS, CPS ratios, and larger cancer diameters than PSMA PET/CT-negative lesions (all *p* < 0.05). Cancer-containing specimens exhibited higher CPS and CPS ratios than benign tissues (median values of 45 vs. 3, and 9.9 vs. 1.0, both *p* < 0.001). In total, 12/13 (92%) patients achieved cBR. Conclusions: This device yielded excellent detection rates with good correlation to preoperative imaging and histological results without adverse events.

## Simple Summary

Salvage surgeries for prostate cancer are a challenging topic in this era of metastasis-directed therapy. Here, we present the first thirteen robot-assisted, prostate-specific membrane antigen-radioguided procedures in patients with oligorecurrent disease, performed using the SENSEI^®^ drop-in gamma probe. The aim of this study was to assess the feasibility and safety of this new device, as well as to evaluate the oncologic outcome based on prostate-specific antigen post-discharge.

## 1. Introduction

With the widespread implementation of prostate-specific membrane antigen (PSMA) positron emission tomography/computed tomography (PET/CT) imaging, the detection of early oligorecurrent PCa lesions at biochemical recurrence has significantly improved [1]. This diagnostic technique has enabled the identification of early recurrent prostate cancer especially at low prostate-specific antigen (PSA) values, opening the possibility for more timely and targeted treatment [2]. Numerous studies explored and continue to investigate the potential of surgically excising metastases as a low-morbidity approach with promising outcomes when compared with radiotherapy (RT) [3,4,5,6,7]. In these procedures, PSMA can act as a targeting tool thanks to radioactively labeled tracers, highlighting PSMA-suspicious lesions which are subsequently removed [8,9]. This entire process falls under the definition of ‘PSMA-radioguided surgery’ (PSMA-RGS) [10]. In recent years, expert surgeons have refined this technique by using miniaturized gamma probes suitable for robotic minimal-invasive surgery [11]. The SENSEI^®^ drop-in gamma probe is a small, single-use device that ensures good maneuverability [12,13]. It offers both numerical and audio feedback through a control unit equipped with a display. Furthermore, the measured radioactivity can be displayed in real-time in the console of the da Vinci Surgical System^®^ using TilePro™ (a multi-input display system; Intuitive Surgical, Sunnyvale, CA, USA) [14,15,16,17]. Recently, the SENSEI^®^ drop-in gamma probe has proven effective for sentinel lymph node dissection during radical prostatectomy (RP) and extended pelvic lymph node dissection (PLND) for PCa [12]. Therefore, we aimed to assess the applicability of this probe in salvage surgeries for oligorecurrent PCa using PSMA-RGS, focusing on its feasibility compared to preoperative PSMA PET/CT imaging and postoperative histological findings, as well as its intraoperative safety during the procedure.

## 2. Materials and Methods

### 2.1. Population and Data Collection

The first 13 consecutive patients who underwent robotic PSMA-RGS using the novel SENSEI^®^ gamma probe (Lightpoint, a Telix company, Amersham, UK) from February-June 2024 were included in this study. All patients initially underwent radical prostatectomy (RP), and pelvic lymph node dissection (PLND) was conducted in 11 out of 13 cases (85%) during the primary treatment. Nodal metastases (pN1) were identified in 2 of these 11 patients (18%) based on the pathology report. Subsequently, they received PSMA PET/CT imaging following biochemical recurrence (BCR), which revealed oligorecurrent PCa (considered as ≤3 lesions following two recent multicentric trials based on MDT) [4,18]. As other inclusion criteria, we stated that each case was considered suitable for the procedure following a multidisciplinary team evaluation, and each patient underwent specific counseling, emphasizing the experimental nature of the study.

As for the exclusion criteria, patients were deemed ineligible in the presence of bone metastasis, prior PSMA-RGS, androgen deprivation therapy (ADT) within 6 months prior to the procedure, an open-approach RGS, or when CPS measurements were performed using different gamma probes (the entire selection process is depicted in Figure S1).

All surgical procedures were performed by an experienced surgeon between February and June 2024 at the Martini-Klinik Prostate Cancer Center, University Hospital Hamburg-Eppendorf. This retrospective analysis has received ethical approval from the local review board (2019-PS-09; PV7316). All men signed an informed consent form on data collection; questionnaires were used for follow-up.

### 2.2. Procedure

**Figure 1:**
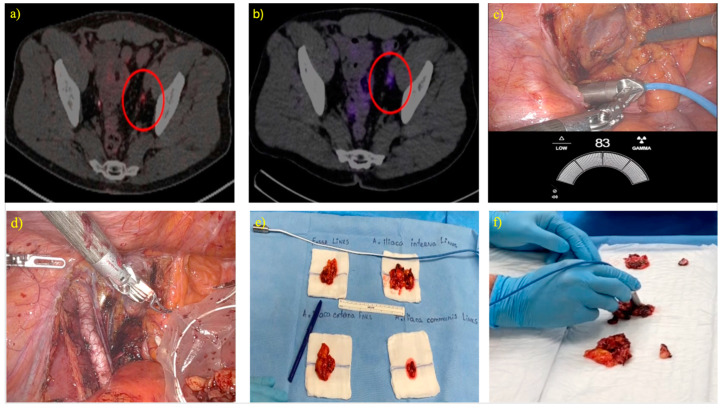
Step-by-step procedure for robot-assisted, radioguided surgery with Lightpoint SENSEI drop-in gamma probe for local recurrences; (**a**) PSMA PET/CT fusion imaging identifies eligibility, (**b**) pre-operative [99mTc]Tc-PSMA-I&S SPECT/CT fusion imaging to cross validate tracer uptake, (**c**) in vivo localization and assessment of the extent of the local recurrence using the Lightpoint SENSEI drop-in gamma probe, (**d**) resection of the suspected metastasis and placement in a retrieval bag, (**e**) placement of all specimens on a separate table with a description of their anatomical site and dimensions, and (**f**) ex vivo measurements of the surgical specimens using the same probe. Abbreviations: PSMA = prostate-specific membrane antigen, PET = positron emission tomography, CT = computed tomography, [^99m^Tc]Tc-PSMA-I&S = ^99m^Technetium PSMA Imaging & Surgery tracer, SPECT = single-photon emission computed tomography.

PSMA-RGS is based on a multi-step approach, as illustrated in Figure 1. The pre-operative phase, thoroughly optimized at our clinic and previously described, involves a multidisciplinary evaluation of PSMA PET/CT fusion imaging to determine eligibility for the procedure (Figure 1a). This is followed by the intravenous injection of [^99m^Tc]Tc-PSMA-I&S, as detailed by Robu et al., one day prior to surgery, and single-photon emission computed tomography/computed tomography (SPECT/CT) for cross validation (Figure 1b) [19]. The maximum and mean standardized uptake values (SUV_max_ and SUV_mean_, respectively) were calculated from a volume of interest around each target lesion with isocontours set at 40% of the maximum. During the procedure, in vivo measurements using the drop-in gamma probe guided the surgeon to the lesion’s location and allowed for the determination of the extent of the local recurrence (Figure 1c). Moreover, as depicted in the same figure, the exact CPS value was displayed live on the screen. The resected tissue specimens were placed in a specimen retrieval bag (Figure 1d). Subsequently, after the removal of all specimens, they were placed on a separate table with a precise description of each specimen’s anatomical site, dimensions, and the maximal CPS value obtained during the procedure (Figure 1e). Finally, CPS quantification was performed ex vivo on the specimens, specifying the maximal value measured, to confirm that the suspicious tissue had been excised (Figure 1f). All surgeries were performed using a DaVinci X robotic platform (Intuitive Surgical, Sunnyvale, CA, USA). The SENSEI gamma probes were provided by Lightpoint, a Telix Pharmaceuticals company.

### 2.3. Evaluation of Parameters and Outcomes of Interest

The radioactivity of each lesion/specimen was measured during the dissection in vivo and ex vivo as the maximum counts per second (CPS). Ex vivo measurements of specimens were performed on the back table in the operating room. The CPS ratio was calculated separately for each patient by dividing the ex vivo maximal CPS of a specimen by the mean of all the ex vivo maximal CPS of specimens without cancerous tissue on final histopathology. If all specimens from one patient contained cancerous material, the mean of all ex vivo maximal CPS values from specimens containing only benign tissue across the entire cohort was applied. The pathology report described the presence of cancer (nodal or soft tissue metastasis), its maximal diameter (d_max_), and the anatomical site of origin. If d_max_ was not reported, the long axis diameters of the respective lesions were measured in preoperative imaging. As an oncological outcome, the PSA was measured within 16 weeks post-surgery to assess complete biochemical response (cBR), defined as PSA < 0.2 ng/mL.

### 2.4. Statistical Analyses

Descriptive statistics included frequencies and proportions for categorical variables. Continuous variables were measured by medians and interquartile ranges (IQR). The Wilcoxon rank sum test and Pearson’s Chi-squared tests examined the statistical significance of differences in medians and proportions, respectively. Scatter plot diagrams were shaped to describe the relationship between intraoperative CPS measurements and specimen characteristics in preoperative imaging (SUV_max_, short axis diameter, localization) and histological examination (cancerous tissue, maximum cancer diameter). All statistical analyses were performed using the R software environment (version 4.3.1; The R Foundation for Statistical Computing, Vienna, Austria). A *p*-value of 0.05 was considered the threshold for statistical significance.

## 3. Results

### 3.1. Overall Cohort

At PSMA-RGS, the median age was 71 (IQR 66–72) years. The median PSA was 0.93 ng/mL (IQR 0.48–1.71) prior to salvage surgery (Table 1). All patients had one lesion on PSMA PET/CT imaging. In 12/13 (92%) patients, these lesions were pelvic. The median long and short axes of the lesions on preoperative PSMA PET/CT were 10.0 mm (IQR 8.0–13.0) and 6.0 mm (IQR 4.0–8.0), respectively, and the median SUV_max_ was 8.4 (IQR 5.5–11.5).

### 3.2. Surgical Parameters

During the surgical procedures, a total of 54 specimens were excised (approximately four specimens per patient). The median blood loss was 100 mL (IQR 35–100) and the median operative time was 134 min (IQR 107–147).

### 3.3. Correlation of Intraoperative Radioactivity Measurements and Preoperative PSMA PET/CT

**Figure 2:**
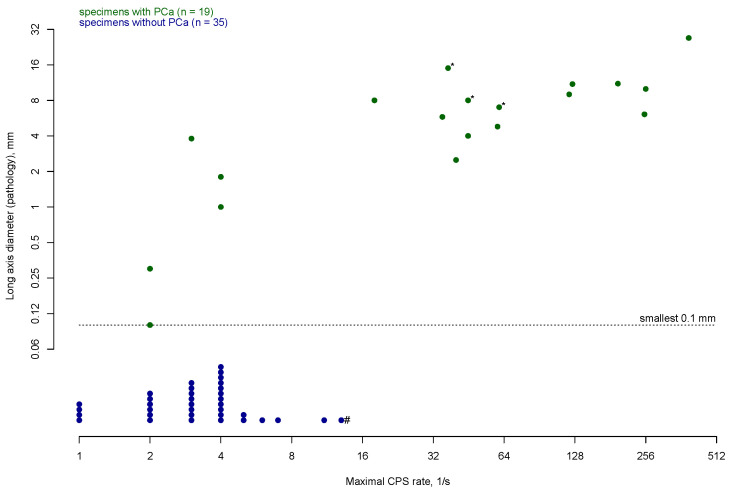
Maximal CPS and long axis diameter (if applicable) of removed specimens during robot-assisted PSMA-radioguided salvage surgery using the SENSEI^®^ gamma probe (n = 54). Results are displayed on a logarithmic scale (to the base 2). PCa = prostate cancer, CPS = counts per second, PSMA = prostate-specific membrane antigen. * Size measurement was not reported for pathologic examination and was derived from pre-operative imaging. # Lymphatic invasion by chronic lymphocytic leukemia was described in pathology report of this specimen.

**Figure 3:**
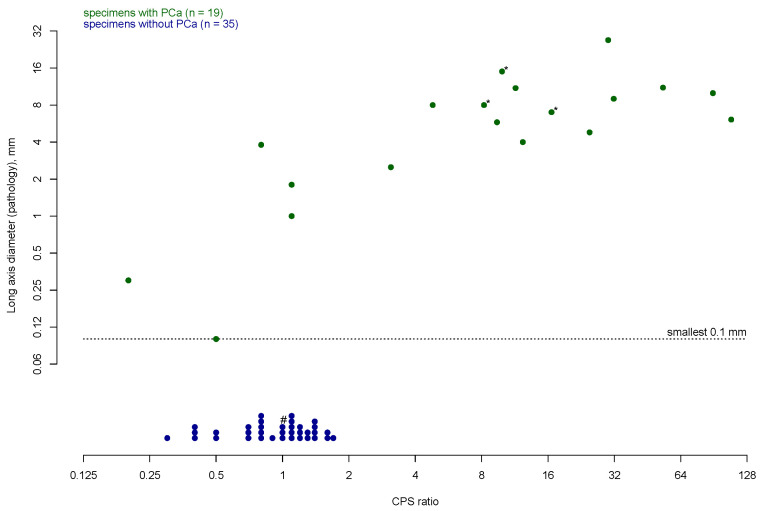
CPS ratio and long axis diameter (if applicable) of removed specimens during robot-assisted PSMA-radioguided salvage surgery using the SENSEI^®^ gamma probe (n = 54). Results are displayed on a logarithmic scale (to the base 2). PCa = prostate cancer, CPS = counts per second, PSMA = prostate-specific membrane antigen. * Size measurement was not reported for pathologic examination and was derived from pre-operative imaging. # Lymphatic invasion by chronic lymphocytic leukemia was described in pathology report of this specimen.

All preoperative PSMA PET/CT-positive lesions were removed, and cancerous tissue was confirmed in all patients (Table 2). The median background radioactivity was at 3.7 CPS (IQR 3.7–4.9). PSMA PET/CT-avid lesions exhibited higher ex vivo CPS than PSMA PET/CT-negative lesions (median 61, IQR 45–195 vs. median 4, IQR 2–4; *p* = 0.02, Figure 2, Table S1). Correspondingly, PSMA PET/CT-avid lesions also had a higher CPS ratio than PSMA PET/CT-negative lesions (median 16.6, IQR 9.9–31.8 vs. median 1.0, IQR 0.6–1.1; *p* < 0.001; Figure 3, Table S1).

### 3.4. General Results of Surgery and Histopathology

**Figure 4:**
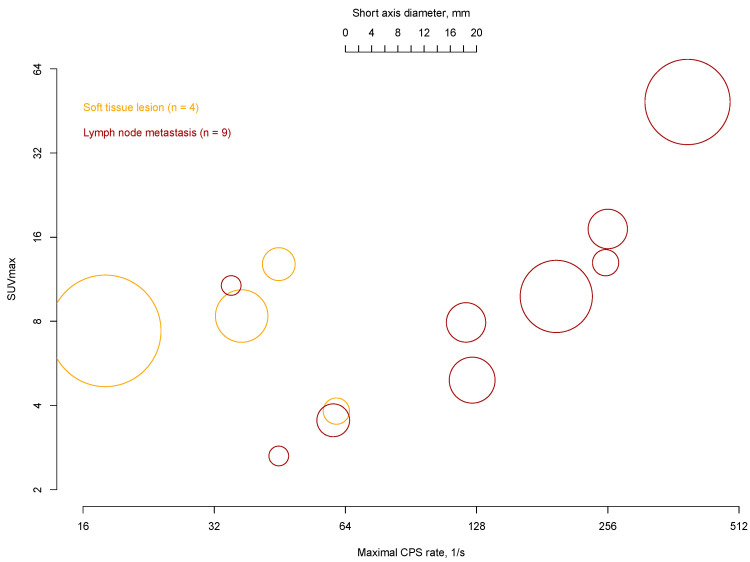
Maximal CPS rates of each primary lesion during robot-assisted PSMA-radioguided salvage surgery using the SENSEI^®^ gamma probe in relation to the tissue type, the SUV_max_, and the short axis diameter measured in preoperative PSMA imaging (n = 13). Results are displayed on a logarithmic scale (to the base 2). The size of each circle represents the long axis diameter in preoperative PSMA/CT imaging (scale at the top). CPS = counts per second, PSMA = prostate-specific membrane antigen, CT = computed tomography.

Upon histopathological evaluation, 19 specimens contained cancer, of which 14/19 (74%) were detected intraoperatively (Table 2 and Table S2). Of these malignant lesions, 9/14 (64%), 4/14 (29%), and 1/14 (7%) were classified as nodal PCa metastases, as soft tissue PCa metastases, and as lymph node infiltration of chronic lymphocytic leukemia, respectively. Six patients had one PCa lesion removed, three patients had two positive lesions, and four patients had more than three PCa lesions. The median d_max_ was 8.0 mm (IQR 6.1–11.0). PCa metastases had a median ex vivo CPS of 45 (IQR 11–123) vs. 3 (IQR 2–4) for benign lesions (*p* < 0.001; Table S3). Cancer lesions detected by in vivo measurements (n = 14) exhibited higher ex vivo CPS (median 61, IQR 41–178 vs. median 3, IQR 2–4; *p* = 0.001; Table S2) and higher CPS ratios (median 14.5, IQR 9.5–31.4 vs. median 0.8, IQR 0.5–1.1; *p* = 0.001); Table S2) compared to in vivo undetected cancer lesions. Considering again the radioactivity detected during the procedure, the median d_max_ of detected vs. undetected lesions was 8.0 mm (IQR 5.9–10.8) vs. 1.0 mm (IQR 0.3–1.8; *p* = 0.002; Table S2). Moreover, cancerous lesions that were positive on preoperative PSMA PET/CT were larger on histopathological examination than PSMA PET/CT-negative PCa manifestations (median 8.0 mm, IQR 6.1–11.50 vs. median 1.4 mm, IQR 0.5–2.3; *p* < 0.001). Of note, soft tissue PCa lesions showed a tendency for lower CPS scores than metastatic lymph node lesions in correlation to their size (Figure 4).

### 3.5. Short-Term Oncological Outcomes and Intraoperative Safety

After PSMA-RGS, 12/13 patients (92%) achieved a complete biochemical response (PSA < 0.2 ng/mL). No adverse events concerning the intraoperative use of the SENSEI^®^ gamma probe or application of [^99m^Tc]Tc-PSMA-I&S were observed.

## 4. Discussion

PSMA-RGS represents an interesting treatment alternative for patients with oligorecurrent PCa to prolong systemic therapy-free survival [20,21]. In particular, the oncological outcomes of this salvage surgery have not yet been clearly defined and are currently being investigated in prospective studies: TRACE-II trial (PSMA-RGS + short-term ADT vs. short-term ADT only- NCT05555017) and PEACE V trial (STORM-NCT03569241) designed for nodal recurrent oligorecurrent PCa [metastasis-directed therapy (MDT surgical or RT) vs. MDT + long-term ADT] [22,23]. As the robot-assisted approach has gained popularity for initial radical prostatectomy, many patients wish to undergo salvage surgery in the same fashion [24]. Therefore, our analysis of the technical possibilities with the novel SENSEI^®^ gamma probe provides several important findings with direct clinical implications. Minimally invasive techniques have been facilitated by significant technological improvements and the introduction of new surgical tools. The latter are usually miniaturized and equipped with highly advanced technology; these two aspects are the foundation of this new SENSEI^®^ drop-in gamma probe, which can be easily inserted through a 12 mm laparoscopic port [25]. The procedures have demonstrated favorable outcomes: all PSMA PET/CT-avid lesions could be detected intraoperatively and were successfully excised, and at least one recurrence was diagnosed in each case. The median blood loss and operative time were consistent with values reported in another recent article (100 mL, IQR 35–100 mL, and 134 min, IQR 107–147) compared to this study’s median of 50 mL, IQR 42–100 mL, and 152 min, IQR 42–100, respectively [11]. The duration of salvage lymph node dissection may be influenced by fibrosis and scarring, which are direct consequences of the primary RP, and subsequent possible adjuvant RT [26,27]. So, tracers may help limit the dissection to the target and surrounding tissues.

No adverse events concerning the intraoperative usage of the SENSEI^®^ gamma probe or the application of [^99m^Tc]Tc-PSMA-I&S were observed. In addition, short-term oncological results were promising, with 92% of patients achieving PSA levels below 0.2 ng/mL, which has been associated with improved treatment-free survival [28]. These encouraging results may justify delaying ADT treatment; however, this therapeutic strategy must be supported by additional PSA measurements below the previously mentioned cutoff [3].

Ex vivo radioactivity offered a more reliable value due to fewer interferences and the comfort of performing the measurement away from the surgical field without background radioactivity; on the other hand, CPS are widely used units of measurement in this field of research and, as much as possible, help compare studies on PSMA-RGS, facilitating their interpretation [29,30]. Moreover, similar to what was reported by Quarta et al., we applied the CPS ratio to adjust for inter-patient differences in background radiotracer uptake [31]. Compared to benign specimens, the ratio was significantly higher in cancerous lesions (median 16.6 vs. median 1.0, *p* < 0.001). Conversely, only five of nineteen cancer recurrences exhibited a CPS ratio lower than two.

Secondly, CPS measurements determined an important correlation with pre-operative PSMA PET/CT imaging. PSMA PET/CT-positive recurrences exhibited higher CPS and CPS ratios than PSMA PET/CT-negative tissue specimens (*p* < 0.001, respectively). In contrast, five metastases were only detected by postoperative histopathological evaluation, constituting 26% of the total. None of these showed increased radioactivity by in and ex vivo gamma probe measurements. It must be noted that the measurement of radioactivity using the gamma probe in vivo may also be impeded by background radioactivity, e.g., due to proximity to the urinary tract. For instance, close to the ureter, bladder, or kidneys, elevated CPS values may be measured due to the high radioactivity of the renally excreted tracer [9,32]. Jilg et al. described that nodal metastases without pathological uptake on PET/CT (false-negative PET/CT lesions) were smaller than those of true-positive lesions [33]. In accordance, PET/CT-negative recurrences in our study had a median long-axis diameter of 1.4 mm. This finding reinforces the earlier observation that lesions smaller than 3 mm are often challenging to detect [29,34]. Other important factors associated with each specimen were the intraoperative detection, assessed in vivo through tracer detection, and tracer uptake evaluated by different SUVs. As might be expected, ex vivo radioactivity measurements correlated with in vivo detection: higher CPS measurements (absolute value and ratio) were more often found in specimens which were detected in vivo (*p* = 0.001). More recently, Berrens et al. reported that SUV_max_ was a positive predictor of ex vivo radioactivity measurements during RGS [35]. Finally, another important parameter considered in this analysis was the size of the tumor on histopathological examination. Through this brief experience, we confirmed that the PET/CT detection rate was more reliable for larger lesions, particularly those measured along the long axis (*p* < 0.001), in the subset of recurrences [36]. Notably, one of the node samples revealed the presence of chronic lymphocytic leukemia, a rare event that has been observed in less than 1% of cases and is likely favored by the increased incidence of concomitant tumors [37,38,39,40].

We shall also argue that the difference in registered CPS counts between soft tissue PCa lesions and nodal metastasis (adjusted for size), where the former usually exhibited lower radioactivity, is challenging to explain. It should be underlined that maximum CPS values are influenced by the distribution of radiotracers within tissue fragments; this could suggest that, in soft tissue metastases, the tumor cells are more dispersed as compared to tumor cells located in lymph nodes.

During the use of the new device, the surgeon did not report any malfunction. The procedures were carried out according to the protocol developed at the Martini Klinik, which has been refined over the years and currently includes more than 600 PSMA-RGS procedures.

The extensive experience in this surgical approach had a pivotal role in augmenting the surgeon’s detection capabilities. As previously mentioned, and detailed in Table S1, PCa recurrences were identified even in PSMA PET/CT-negative lesions, where the decision to pause, closely examine the area, and perform precise radioactivity measurements often relies on a high level of expertise. This meticulous approach is particularly critical in cases where the tissue under examination appears suspicious, often exhibiting very limited extension, despite the lack of clear pathological uptake.

Despite its novelty, our study has some limitations. This study’s limited sample size from a single-center patient cohort and its retrospective design restricts the generalizability of the findings. The definition of oligorecurrent prostate cancer (PCa) patients suitable for MDT, based on the number and location of lesions, is not consistent in the literature, limiting the ability to compare outcomes across studies. Furthermore, there is currently no information available on the cost-effectiveness of this probe relative to similar devices. This drop-in gamma probe is single-use, which results in an environmental footprint that is not entirely clear now, but it may be lower than expected, as recently demonstrated for other single-use urological devices [41]. Finally, the oncological outcome was based on a short-term follow-up with PSA measurements taken within 16 weeks after surgery.

The lack of additional postoperative PSA measurements precludes the calculation of biochemical-free survival. Furthermore, any evaluation of delays in ADT treatment, a primary goal of PSMA-RGS, was not feasible due to the recent nature of the treatment. The choice regarding pharmacological therapy is still supported by experimental data from trials with small sample sizes; for this reason, the introduction and timing of ADT post-MDT still lack clear guidelines supported by expert consensus [42,43]. With further follow-up, it may be possible to determine if, in some rare cases, the procedure has been curative, achieving a durable cBR over time [44,45]. It should also be noted that PSMA-guided RT has shown excellent results in the treatment of these metastases and could be a valid therapeutic strategy in the treatment of patients with oligorecurrent PCa, even in the case of bone metastases [46,47]. However, the SENSEI^®^ gamma probe proved to be suitable for robotic surgical RGS, aligning with recent findings published by Harke et al. on the effectiveness of this device in screening for lymph node metastases during robot-assisted RP for primary PCa [48]. Additionally, the integration within the TilePro™ system of the da Vinci platform facilitates the intraoperative monitoring of radioactivity measurements by both surgeons and assistants. This functionality may also enable three-dimensional reconstructions in the future and may pave the way for the potential application of artificial intelligence during the procedure [49,50,51,52]. We hope that this experimental treatment for this subset of PCa patients will be performed exclusively in specialized centers, ensuring expertise and enabling surgeons to refine their techniques. This will include, for instance, the detailed information on feedback and the maneuverability of various probes, optimizing their clinical application and improving surgical precision.

## 5. Conclusions

In this initial case series, the SENSEI^®^ drop-in gamma probe yielded excellent detection rates with robust correlation between intraoperative measurements and pre- and postoperative findings. Further prospective studies with larger cohorts are needed to confirm the results of our analysis, evaluate the cost-effectiveness of this technology, and refine the role of salvage surgery in the management of oligorecurrent PCa over extended follow-up periods. Moreover, in the coming years, this device could be compared with other established technologies available on the market, aiding in the identification of key similarities and differences.

## Figures and Tables

**Table 1:** Baseline characteristics of 13 patients treated with robot-assisted PSMA-radioguided salvage surgery using the SENSEI^®^ gamma probe.

		Overall (*n* = 13)
Age at PSMA-RGS, years	Median [IQR]	71 [66, 72]
PSA prior PSMA-RGS, ng/mL	Median [IQR]	0.93 [0.48, 1.71]
No. of PSMA PET/CT avid lesions	n, %	
One		13 (100%)
Localization of PSMA PET/CT avid lesions	n, %	
Pelvic		12 (92.3%)
Extra-pelvic		1 (7.7%)
Time RP to PSMA-RGS, months	Median [IQR]	49 [25, 70]
Prior radiotherapy	n, %	5 (38.5%)
SUV_max_ *	Median [IQR]	8.4 [4.9, 12.8]
SUV_mean_ *	Median [IQR]	5.5 [3.8, 6.8]
Long axis diameter (on CT) *	Median [IQR]	10.0 [8.0, 13.0]
Short axis diameter (on CT) *	Median [IQR]	6.0 [4.0, 8.0]

* Measurement corresponds to the primary lesion per patient. Abbreviations: CT = computed tomography, PSA = prostate-specific antigen, PSMA = prostate-specific membrane antigen, PET = positron emission tomography, IQR = interquartile range, NA = not assigned, RP = radical prostatectomy, SUV = standard uptake value, RGS = radioguided surgery.

**Table 2:** Results of 13 patients treated with robot-assisted PSMA-radioguided salvage surgery using the SENSEI^®^ gamma probe.

		Overall (*n* = 13)
No. of pathologically positive lesions	n, %	
Negative		0 (0%)
1		6 (46.2%)
2		3 (23.1%)
≥3		4 (30.8%)
Long axis diameter (pathology) *	Median [IQR]	8.0 [6.1, 11.0]
CPS background measurement **	Median [IQR]	3.7 [3.7, 4.9]
Complete biochemical response	n, %	
Yes		12 (92.3%)
No		1 (7.7%)
Soft tissue lesions ***	n, %	4 (30.8%)

* Measurement corresponds to the primary lesion per patient. In three patients, where no size was measured during pathology examination, we assigned the diameter based on preoperative imaging. All patients with soft tissue lesions did not harbor any additional lymph node metastases. ** CPS background was defined as average CPS of specimens without cancerous tissue at a patient level. If all specimens of one patient contained cancerous tissue (n = 3), the average of all negative specimens within the cohort was used. *** Soft tissue lesions that were not lymph node metastases. Abbreviations: CPS = counts per second, IQR = interquartile range, PSMA = prostate-specific membrane antigen.

## Data Availability

The data presented in this study are available on request from the corresponding author. The data are not publicly available due to ethical reasons.

## Supplementary Materials

The following supporting information can be downloaded at: https://www.mdpi.com/article/10.3390/cancers17010093/s1, Figure S1: CONSORT flow diagram illustrating the patient selection process for robotic PSMA-RGS using the SENSEI® drop-in gamma probe; Table S1: Subgroup analyses for specimens with cancer (n = 19): PSMA PET and max. ex vivo CPS, CPS ratio, LAD; Table S2. Subgroup analyses for specimens with cancer (n = 19): Detection intraoperative in vivo and max. ex vivo CPS, CPS ratio, LAD; Table S3. Analyses of all specimens: Histology and max. ex vivo CPS, CPS ratio, LAD.
