# Supplementary material for: Robot-Assisted PSMA-Radioguided Salvage Surgery for Oligorecurrent Prostate Cancer Using the Novel SENSEI® Drop-in Gamma Probe: Correlation of Intraoperative Measurements to Preoperative Imaging and Final Histology
Source: Cancers (Basel). 2024 Dec 31;17(1):93. doi: 10.3390/cancers17010093 (PMC11720234; doi:10.3390/cancers17010093)
Supplement: Supplementary file 1 [file cancers-17-00093-s001.zip › cancers-3354239-supplementary.pdf]

Table S1. Subgroup analyses for specimens with cancer (n=19): PSMA PET and max. *ex vivo* CPS, CPS ratio, LAD.

		**PSMA PET/CT positive** ***(n=13)***	**PSMA PET/CT negative** ***(n=6)***	***p***
**CPS**	*Median [IQR]*	61 [45,195]	4 [2,4]	0.002
**CPS ratio**	*Median [IQR]*	16.6 [9.9,31.8]	1.0 [0.6,1.1]	<0.001
**LAD**	*Median [IQR]*	8.0 [6.1,11.0]	1.4 [0.5, 2.3]	<0.001

Abbreviations: CPS = counts per second, IQR = interquartile range, PSMA = prostate-specific membrane antigen, PET = positron emission tomography, CT = computed tomography, LAD = long axis diameter of the lesion (as indicated in the pathology report).

Table S2. Subgroup analyses for specimens with cancer (n=19): Detection intraoperative *in vivo* and max. *ex vivo* CPS, CPS ratio, LAD.

		**Detection intraoperative *in vivo* positive** ***(n=14)***	**Detection intraoperative *in vivo* negative** ***(n=5)***	***p***
**CPS**	*Median [IQR]*	61 [41, 178]	3 [2, 4]	0.001
**CPS ratio**	*Median [IQR]*	14.5 [9.5,31.4]	0.8 [0.5,1.1]	0.001
**LAD**	*Median [IQR]*	8.0 [5.9,10.8]	1.0 [0.3, 1.8]	0.002

Abbreviations: CPS = counts per second, IQR = interquartile range, LAD = long axis diameter of the lesion (as indicated in the pathology report).

Table S3. Analyses of all specimens: Histology and max. *ex vivo* CPS, CPS ratio, LAD.

		**Histology positive** ***(n=19)***	**Histology negative** ***(n=35)***	***p***
**CPS**	*Median [IQR]*	45 [11,123]	3 [2, 4]	<0.001
**CPS ratio**	*Median [IQR]*	9.9 [2.1,27.4]	1.0 [0.8,1.3]	<0.001
**LAD**	*Median [IQR]*	6.1 [3.2, 9.5]	NA	

Abbreviations: CPS = counts per second, IQR = interquartile range, LAD = long axis diameter of the lesion (as indicated in the pathology report).

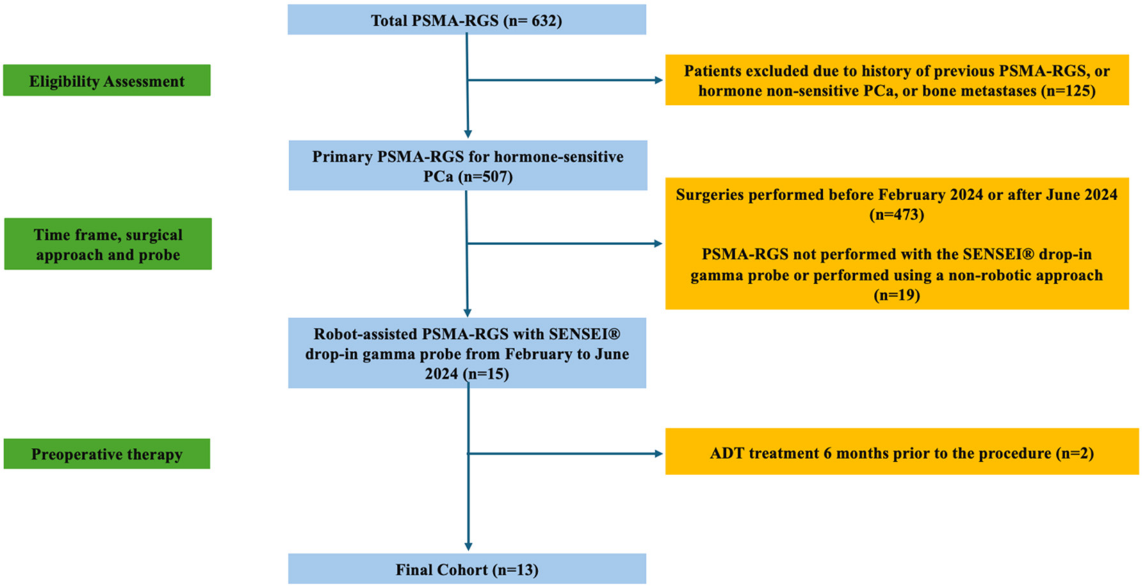

Figure S1. CONSORT flow diagram illustrating the patient selection process for robotic PSMA-RGS using the SENSEI® drop-in gamma probe. The inclusion and exclusion criteria are detailed, resulting in a final cohort of 13 patients. A total of 19 surgical specimens were excised, with pathological confirmation of recurrent prostate cancer. Blue boxes represent the stepwise progression of patient cohorts during the process based on inclusion and exclusion criteria; green boxes highlight the main selection criteria applied at each stage; yellow boxes indicate the groups of excluded patients along with specific reasons at each selection step. Abbreviations: PSMA = prostate-specific membrane antigen, PET = positron emission tomography, CT = computed tomography, RGS = radioguided surgery, PCa = prostate cancer, ADT = androgen deprivation therapy.
